# Direct Whole-Genome Sequencing of Sputum Accurately Identifies Drug-Resistant Mycobacterium tuberculosis Faster than MGIT Culture Sequencing

**DOI:** 10.1128/JCM.00666-18

**Published:** 2018-07-26

**Authors:** Ronan M. Doyle, Carrie Burgess, Rachel Williams, Rebecca Gorton, Helen Booth, James Brown, Josephine M. Bryant, Jackie Chan, Dean Creer, Jolyon Holdstock, Heinke Kunst, Stefan Lozewicz, Gareth Platt, Erika Yara Romero, Graham Speight, Simon Tiberi, Ibrahim Abubakar, Marc Lipman, Timothy D. McHugh, Judith Breuer

**Affiliations:** a Division of Infection and Immunity, University College London, London, United Kingdom; b Microbiology, Virology and Infection Control, Great Ormond Street Hospital NHS Foundation Trust, London, United Kingdom; c Centre for Clinical Microbiology, Division of Infection and Immunity, Royal Free Campus, UCL, London, United Kingdom; d University College London Hospitals NHS Foundation Trust, London, United Kingdom; e North Central London TB Service-South Hub, Whittington Hospital NHS Trust, London, United Kingdom; f Royal Free London NHS Foundation Trust, London, United Kingdom; g Molecular Immunity Unit, MRC Laboratory of Molecular Biology, Department of Medicine, University of Cambridge, Cambridge, United Kingdom; h Oxford Gene Technology, Oxford Begbroke Science Park, Begbroke, Oxfordshire, United Kingdom; i Blizard Institute, Barts and The London School of Medicine and Dentistry, Queen Mary University of London, London, United Kingdom; j North Middlesex University Hospital NHS Trust, London, United Kingdom; k UCL Institute for Global Health, University College London, London, United Kingdom; l UCL Respiratory, Division of Medicine, University College London, London, United Kingdom; University Hospital Münster

**Keywords:** Mycobacterium tuberculosis, whole-genome sequencing, pathogen DNA enrichment, antimicrobial resistance

## Abstract

The current methods available to diagnose antimicrobial-resistant Mycobacterium tuberculosis infections require a positive culture or only test a limited number of resistance-associated mutations. A rapid accurate identification of antimicrobial resistance enables the prompt initiation of effective treatment. Here, we determine the utility of whole-genome sequencing (WGS) of M. tuberculosis directly from routinely obtained diagnostic sputum samples to provide a comprehensive resistance profile compared to that from mycobacterial growth indicator tube (MGIT) WGS. We sequenced M. tuberculosis from 43 sputum samples by targeted DNA enrichment using the Agilent SureSelectXT kit, and 43 MGIT positive samples from each participant. Thirty two (74%) sputum samples and 43 (100%) MGIT samples generated whole genomes. The times to antimicrobial resistance profiles and concordance were compared with Xpert MTB/RIF and phenotypic resistance testing from cultures of the same samples. Antibiotic susceptibility could be predicted from WGS of sputum within 5 days of sample receipt and up to 24 days earlier than WGS from MGIT culture and up to 31 days earlier than phenotypic testing. Direct sputum results could be reduced to 3 days with faster hybridization and if only regions encoding drug resistance are sequenced. We show that direct sputum sequencing has the potential to provide comprehensive resistance detection significantly faster than MGIT whole-genome sequencing or phenotypic testing of resistance from cultures in a clinical setting. This improved turnaround time enables prompt appropriate treatment with associated patient and health service benefits. Improvements in sample preparation are necessary to ensure comparable sensitivities and complete resistance profile predictions in all cases.

## Abstract (precis)

The current methods available to diagnose antimicrobial-resistant Mycobacterium tuberculosis infections require a positive culture or only test a limited number of resistance-associated mutations. A rapid accurate identification of antimicrobial resistance enables the prompt initiation of effective treatment.

## INTRODUCTION

Tuberculosis (TB) infection is a global emergency associated with an increasing burden of drug-resistant Mycobacterium tuberculosis complex infections (1). Phenotypic testing for antimicrobial resistance detection is slow, with results typically a month to 6 weeks after initial culture confirmation, leading to the potential for prolonged suboptimal antibiotic treatment. Molecular assays such as the Xpert MTB/RIF (Cepheid), MTBDRplus, and MTBDRsl (Hain Lifescience) can rapidly detect a limited number of first- and second-line drug resistance mutations (2). However, none are currently able to identify the full range of antibiotic resistance mutations needed for appropriately targeted therapy in people with multidrug-resistant (MDR) TB. Further, these assays recognize only a fixed number of target mutations, missing less common resistance mutations (3), while Xpert MTB/RIF can only detect DNA mutations and cannot predict amino acid changes, resulting in potential false positives (4).

Whole-genome sequencing (WGS) of M. tuberculosis enables a comprehensive identification of all known drug-resistant mutations for all classes of TB drugs and also can provide valuable contact tracing information (5). Recently, the sequencing of organisms cultured in mycobacterial growth indicator tubes (MGITs) has been shown to be both an accurate method for detecting first- and second-line resistance mutations across the genome and less expensive than present routine diagnostic workflows (6). Although it is being rolled out in England (7), it relies on bacterial culture, which can delay the time to result by several weeks.

We previously described a successful method for capturing M. tuberculosis DNA directly from sputum samples using biotinylated RNA baits (8). This protocol provides a possible faster alternative to sequencing M. tuberculosis whole genomes and could therefore offer a quicker diagnosis of antibiotic resistance, leading to tailored treatment regimens with less use of antimicrobials and associated toxicity, fewer days in the hospital, reduced cost, and improved outcomes.

Mixed-strain infections of M. tuberculosis are well documented (9) and may lead to poor treatment outcomes and the possible emergence of minority drug-resistant strains (10–12). Culture of M. tuberculosis is known to impact negatively the detection of mixtures and minority variant mutations (13), with short-term MGIT culture being particularly poor at identifying mixed infections (14).

The aims of this study were (i) to compare the utility of performing WGS directly from routinely obtained diagnostic sputum with that from MGIT samples taken from the same participant (time to diagnosis plus their ability to predict antimicrobial resistance [AMR]) and (ii) to identify mixed infections and minority populations within samples.

## MATERIALS AND METHODS

### Study recruitment.

Individuals aged 16 years or older attending a TB service with suspected pulmonary TB at seven clinics in London, UK, were invited to take part in this study.

### DNA extraction.

DNA was extracted from 1-ml clinical samples and MGIT cultures using mechanical ribolysis and an automated DNA extraction workflow. Samples were centrifuged for 30 min at 16,200 × *g*, and the supernatant was discarded. For MGIT cultures only, a saline prewash method was utilized to reduce the human nucleic acid component of the sample (15). One milliliter of sterile saline was added to the pellet (0.9% [wt/vol]), and the pellet was resuspended and centrifuged for 15 min at maximal speed (16,200 × *g*). The supernatant was discarded and the process was repeated. For sputum and MGIT cultures, approximately 50 μl of glass beads (425 to 600 μm) was added to each sample pellet, and ribolysis was performed on a FastPrep24 platform for 45 s at 6.4 m/s. Two hundred forty microliters of extraction buffer 2 and 10 μl of proteinase K were added to each of the samples, which were vortexed and then incubated at 56°C for 10 min. DNA was extracted from samples lysates on the Diasorin IXT (Arrow) automated platform using DNA extraction cartridges eluting into 100 μl.

### Quantification of extracted Mycobacterium tuberculosis DNA.

The Xpert MTB/RIF (Cepheid) assay was performed on sputum samples as per the manufacturer's instructions; the M. tuberculosis quantity was reported as either very low, low, medium, or high alongside threshold cycle (*C_T_*) values. The Xpert MTB/RIF assay also reported rifampin resistance as “detected” or “not detected.” Drug susceptibility testing was based on phenotypic cultures for first-line drugs on solid media using the resistance ratio method and was carried out by the National Mycobacterium Reference Service using their standard protocols. A second M. tuberculosis-specific quantitative PCR (qPCR) targeting the 16S rRNA gene (*rrs*) was utilized to quantify the M. tuberculosis DNA extracted from sputum samples and MGIT cultures. For MGIT culture extracts, a 1/1,000 dilution was prepared prior to qPCR analysis. qPCR was performed using forward primer 5′-GTGATCTGCCCTGCACCTC-3′ and reverse primer 5′-ATCCCACACCGCTAAAGCG-3′ with a TaqMan probe ROX-AGGACCACGGGATGCATGTCTTGT-BHQ2 (16). The M. tuberculosis-specific qPCR mixtures consisted of 12.5 μl of Quantitect Multiplex NoROX mix (Qiagen), 0.2 μM primers and probes, and 5 μl template per reaction mixture in a total volume of 25 μl. Reactions were performed in duplicates on a Rotor-gene 8000 platform. The PCR cycling conditions were as follows: 50°C for 30 min, 95°C for 15 min, and 40 cycles of 94°C for 45 s and 60°C for 45 s. Standards were prepared from commercially sourced M. tuberculosis genomic DNA (Vircell), reconstituted as directed by the manufacturer.

### Sequencing library preparation and whole-genome sequencing.

Total DNA was quantified in sputum and MGIT extracts using the Qubit high sensitivity DNA assay (Life Technologies). Carrier human genomic DNA (Promega) was added where needed to obtain a total of 200 ng of DNA input for library preparation. All DNA samples were sheared using a Covaris S2 ultrasonicator for 150 s (peak incident power [PIP], 175; duty factor, 5; 200 cycles per burst using frequency sweeping). Sputum samples were prepared using the SureSelectXT target enrichment system for the Illumina paired-end sequencing library protocol (Agilent Technologies). End repair, the 3′ addition of adenosine, and the ligation of adapters were all carried out according to Agilent's protocol. Prior to hybridization, 12 cycles of precapture PCR were performed using primers provided in the SureSelectXT kit. Hybridization of M. tuberculosis DNA to the streptavidin-coated beads was carried out using am M. tuberculosis-specific bait set described previously (8). Briefly, 120-mer RNA baits were designed to provide nonredundant coverage of the entire length of the positive strand of the H37Rv reference genome; they were synthesized by Agilent Biotechnologies. The baits can be purchased from Agilent and the bait sequences are available upon request from the authors. Eighteen cycles of postcapture PCR were performed with indexing primers provided in the SureSelectXT kit. All Agilent-recommended quality control steps were carried out. To compare the effect of target enrichment on MGIT sequencing, the first 14 MGIT samples underwent library preparation using SureSelectXT, and all subsequent MGIT samples had DNA libraries prepared using the NEBNext Ultra II DNA Library Prep kit (NEB) as per the manufacturer's protocol. The resulting DNA libraries were run on either a MiSeq or NextSeq sequencer (Illumina) using either a V2 500-cycle or 500/550 Mid output 300-cycle kit, respectively.

### Optimized sequencing method.

Three samples were prepared using a SureSelectXT fast target enrichment system (Agilent Technologies) as per the manufacturer's protocol. A reduced bait set was designed to capture only genes associated with drug resistance, and information for spoligotyping was used in the hybridization step. The reduced set of 120-mer RNA baits was synthesized by Agilent Technologies in the same way as the full set, except that it only included baits that were complementary to the genes and regions in Table 1 of the H37Rv reference genome.

**TABLE 1:** Target genes/regions and reason for inclusion in the reduced M. tuberculosis bait set

Gene target and/or region	Region property
*gyrB/gyrA*	Fluoroquinolone resistance
*rpoB/rpoC*	Rifampin resistance
*rpsL*	Streptomycin resistance
*rrs*	Streptomycin, amikacin, and kanamycin resistance
*gidB*	Streptomycin resistance
*mabA-fabG1, inhA*, and promoter	Isoniazid and ethionamide resistance
*katG*	Isoniazid resistance
*kasA*	Isoniazid resistance
*aphC-oxyR*	Isoniazid resistance
*tlyA*	Capreomycin resistance
*pncA* and promoter	Pyrazinamide resistance
*eis* and promoter	Kanamycin resistance
*thyA*	PAS resistance
*embC*	Ethambutol resistance
*embB*	Ethambutol resistance
*ethA*	Ethionamide resistance
Direct repeat locus	Spoligotyping

### Bioinformatics analysis.

Sequencing reads weretrimmed for adapter content and quality using Trim Galore, keeping reads longer than 100 bp. Trimmed reads were deduplicated and mapped to the H37Rv (accession number NC_000962) reference genome using BBmap, allowing only successfully mapped paired reads at the 99% equivalent minimum identity across the entire read and a maximum insert size of 500 bp. Duplicate mapped reads were removed using Picard tools, and variants against the reference genome were called with freebayes, keeping only variants with a minimum of 10 supporting reads, greater than 2% frequency, mapping quality greater than 20, and a base quality score greater than 30, with reads present on both the forward and reverse strands and on both the 5′ and 3′ ends of reads. Variants found in and within 100 bp of Pro-Glu (PE) and Pro-Pro-Glu (PPE) genes, mobile elements, and repeat regions were discarded. For resistance calling, single nucleotide variants (SNVs) were annotated using ANNOVAR (17). A maximum likelihood phylogeny was also inferred from 1,113 core genome SNVs present in 64 samples representing 32 participants using RAxML (v. 8.2.1) (18) with 99 bootstrap replicates. The SNV distance between pairs was calculated using R package seqinr. The same filtering conditions were also applied to the variants for the minor variant analysis. The number of reads across each variant position was normalized between pairs of samples from the same patient to adjust for the effect of read depth on variant frequency. Minor variants were filtered from the data set if found on reads with greater sequence identity to a different M. tuberculosis complex species. To further control possible contamination of paired samples with low frequency variants, MGIT and sputum samples from each pair were extracted on separate days, prepared in separate sequencing libraries, and sequenced on different runs.

### Ethics approval and consent to participate.

Samples were collected with informed consent from patients attending a TB clinic setting at the participating hospitals. Approval for the study was granted by the NRES Committee East Midlands–Nottingham 1 (REC reference 15/EM/0091). All samples were pseudoanonymized and allocated unique identification numbers.

### Data availability.

All sequence data associated with this study have been deposited in the European Nucleotide Archive under study accession number PRJEB21685.

## RESULTS

### Genomic coverage.

**FIG 1:**
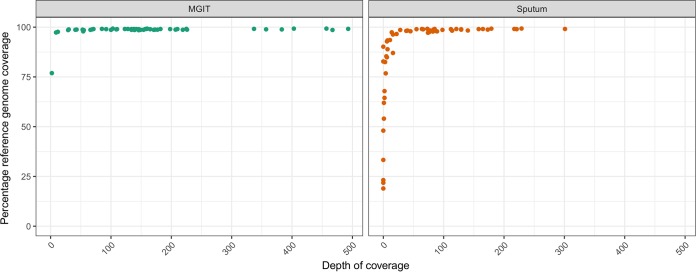
Comparison of the percentages of reference genomes with at least one sequence read covering a position on the *y* axis by the median depth of coverage on the *x* axis for each individual sample. This is stratified by whether original sample material was a sputum or MGIT culture.

Sixty-three participants were prospectively enrolled. Paired sputum and MGIT samples were sequenced from 43 patients. This is due to 10 participants not having an MGIT sample collected for sequencing, another eight samples being smear and Xpert MTB/RIF negative, and two where the volume of sputum was insufficient for DNA extraction. Samples sequenced from MGIT cultures had a higher reference genome coverage than those obtained directly from sputum (Fig. 1), and this was correlated to the increased M. tuberculosis DNA available from the former (see Fig. S1 in the supplemental material). The enrichment of MGIT culture samples using the M. tuberculosis probes also enhanced the quality and depth of the sequences (Fig. S1).

**FIG 2:**
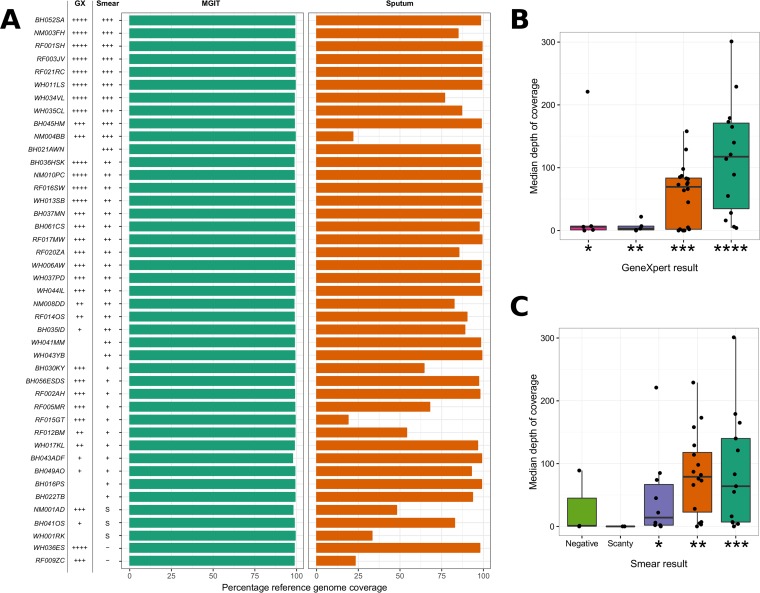
(A) Bar plot showing the percentages of reference genome coverage (a single read covering each genome position) for patients with both sputum and MGIT samples sequenced. The plot is annotated with both Xpert MTB/RIF (GX) and smear microscopy (Smear) results. For GX: ****, high; ***, medium; **, low; *, very low. For smear: ***, 3+; **, 2+; *, 1+; S, scanty; −, negative. Where a result is missing, the test was not carried out. Boxplots showing median depths of coverage for sputum samples stratified by both the quantitative Xpert MTB/RIF measures (B) and semiquantitative smear results (C).

We next evaluated whether bacterial load, as measured by smear and Xpert MTB/RIF, could be used to predict the success of whole-genome sequencing. From 43 patients, 32 sputum samples (74.4%) and 43 MGIT samples (100%) generated whole genomes (>85% coverage against reference genome) (Fig. 2A). Sputum sequencing success was linked to estimated input pathogen copy number. We stratified participants into 16 with high (3+ smear result, Xpert MTB/RIF high), 18 with medium (2+ smear result, Xpert MTB/RIF medium), and 9 with low (scanty or 1+ smear result, Xpert MTB/RIF low) bacterial loads and found that 87.5% of sputum samples with high bacterial load samples generated complete genomes compared to 72.2% with medium and 55.5% with low bacterial loads (Fig. 2B and C). We were also able to recover partial genomes for two sputum samples that were reported as negative by smear microscopy but Xpert MTB/RIF positive.

### MGIT and sputum sequence variation.

**FIG 3:**
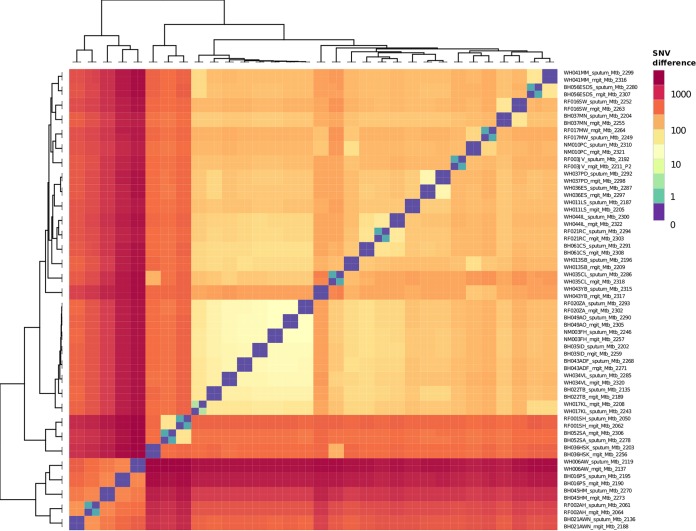
Heatmap clustering samples by the pairwise numbers of single nucleotide differences between them. Sample names are formatted such that the patient identifier is at the start followed by whether the sample originated from a MGIT culture or sputum.

A comparison of the 32 patients with both complete sputum and MGIT genomes available showed no unique consensus sequence variation between the pairs. The identity between sputum and MGIT consensus sequences is shown in a heat map (Fig. 3) and phylogenetic tree (see Fig. S2). Twenty-three MGIT and sputum pairs showed no SNV between them at the consensus level, while nine patients' sample pairs differed by one or two single nucleotides. In all nine patients, the consensus polymorphism was present as a minority variant in the matched sputum or MGIT sample (see Table S1).

### Time to antibiotic resistance prediction.

**FIG 4:**
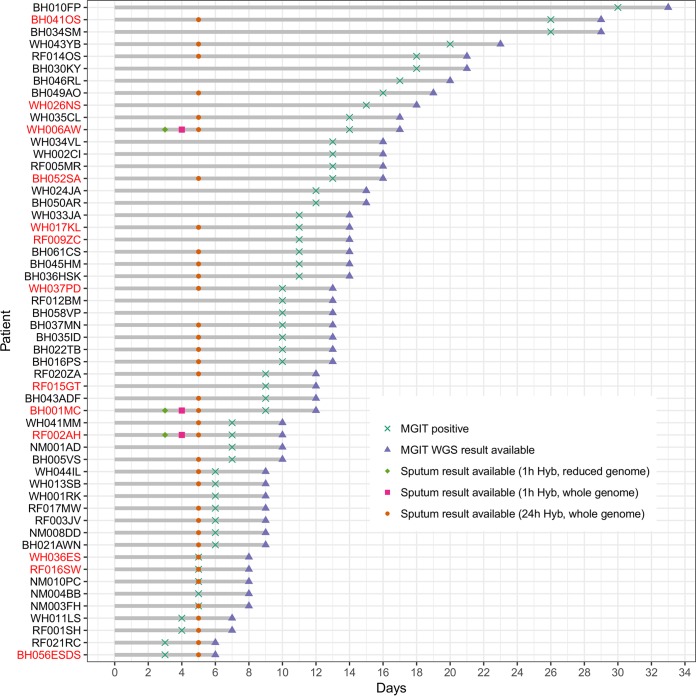
The time taken in days on the *x* axis from sample collection (day 0) to when the MGIT samples flag as positive and sequence results become available, as denoted by the gray bars. The time taken for a patient sputum result to become available is marked by three different identifiers depending on whether the sample underwent whole-genome sequencing or partial-genome sequencing and whether the 24-h or 1-h hybridization protocol was used. Sputum samples that failed sequencing are marked by missing symbols. Patient identifiers marked in red had confirmed drug-resistant TB infections.

Allowing for sample batching, which was only carried out for study purposes, the direct sequencing of sputum using targeted enrichment reduced the time to antibiotic susceptibility prediction initially to 5 days, compared with a mean of 11 (standard deviation [SD], 6) days for MGIT sequencing (Fig. 4). This was reduced further by protocol optimization to 3 days when a reduced bait set was used that captures only the regions with putative resistance mutations (Table 1 and Fig. 4). Hybridization optimization also reduced the whole-genome protocol to 4 days (Fig. 4). The reduced bait set targeted 35,960 bp of the H37Rv reference genome in total and successfully resequenced three MDR-TB samples (noted in Fig. 4.) from this study to a high average depth of coverage (>2,000×) over the captured regions. All eight genotypic resistant variants identified in the whole-genome sequencing data were also identified after resequencing with the reduced bait set (see Fig. S3). Overall, 36 sputum samples with complete genomes, including 77% of those with drug resistance mutations, would have been reported a mean of 9 days earlier than with MGIT sequencing and a mean of 35 days earlier than with phenotypic testing using the optimized 3-day protocol.

### Antibiotic resistance concordance.

We found complete concordance between resistance mutations identified in paired MGIT and sputum samples from nine participants when there was >85% single read coverage against the reference genome. Four participants missed resistance mutations where sputum sequencing read coverage was too low to make a reliable call (Table 2). Xpert MTB/RIF, sputum WGS, and MGIT WGS were concordant with phenotypic resistance testing in 21 of 23 resistance mutations identified (Table 2). The exceptions were where a variant in participant RF015GT predicting an amino acid change Ser428Iso in *rpoB* (H37Rv codon numbering; *Escherichia coli rpoB* numbering S509I) was reported as resistant by Xpert MTB/RIF, but the reference laboratory found it to be susceptible (Table 2). Previous publications have shown that not all SNVs at this position are associated with resistance (19–21). A fixed mutation in *katG* predicting amino acid change Ser315Thr was confirmed to confer high levels of resistance to isoniazid in five samples, but patient BH052SA with the same mutation was found to be susceptible. This common polymorphism in *katG* has been previously been shown to confer consistently high levels of isoniazid resistance to M. tuberculosis (22–24). Whole-genome sequencing from both MGIT and sputum samples also identified streptomycin (three patients) and *p*-aminosalicylic acid ([PAS] four patients) resistance mutations, neither of which is routinely tested within the phenotypic assay in the United Kingdom.

**TABLE 2:** Antimicrobial resistance profiles from 13 patients with evidence of resistance from direct sputum sequencing using whole-genome bait set

Patient	Smear result	Rifampicin								Isoniazid							Ethambutol					Pyrazinamide			Streptomycin					PAS		
		*rpoB*:c.C1333T:p.H445Y		*rpoB*:c.1333_1334TG		*rpoB*:c.C1349T:p.S450L		GeneXpert RIF resistance	Solid culture phenotype	*katG*:c.G944C:p.S315T		*fabG1*:c.-8		*inhA*:c.T280G:p.S94A		Solid culture phenotype	*embB*:c.G1217A:p.G406D		*embB*:c.A1490G:p.Q497R		Solid culture phenotype	*pncA*:c.T11C:p.L4S		Solid culture phenotype	*rpsL*:c.A128G:p.K43R		gid:c.102delG:p.G34fs		Solid culture phenotype	*thyA*:c.A604G:p.T202A		Solid culture phenotype
		Sputum	MGIT	Sputum	MGIT	Sputum	MGIT			Sputum	MGIT	Sputum	MGIT	Sputum	MGIT		Sputum	MGIT	Sputum	MGIT		Sputum	MGIT		Sputum	MGIT	Sputum	MGIT		Sputum	MGIT	
BH001MC	+	S^*a*^	ND^*b*^	S	ND	S	ND	ND	S	S	ND	S	ND	S	ND	S	S	ND	S	ND	S	ND	S	S	S	ND	S	ND	ND	R	ND	ND
BH041OS	+	—^*c*^	S	—	S	—	R^*d*^	R	R	—	R	—	S	—	S	R	—	S	—	R	R	—	R	R	—	R	—	S	ND	—	S	ND
BH052SA	+++	S	S	S	S	S	S	S	S	R	R	S	S	S	S	S	S	S	S	S	S	S	S	S	S	S	S	S	ND	S	S	ND
BH056ESDS	+	S	S	S	S	S	S	S	S	S	S	S	S	S	S	S	S	S	S	S	S	S	S	S	S	S	S	S	ND	R	R	ND
RF002AH	+	S	S	S	S	S	S	ND	S	R	R	S	S	S	S	R	S	S	S	S	S	S	S	S	S	S	S	S	ND	S	S	ND
RF009ZC	Neg	—	S	—	S	—	S	S	S	—	R	—	S	—	S	R	—	S	—	S	S	—	S	S	—	R	—	S	ND	—	S	ND
RF015GT	+	—	S	—	S	—	S	R	S	—	S	—	S	—	S	S	—	S	—	S	S	—	S	S	—	S	—	S	ND	—	S	ND
RF016SW	++	S	S	S	S	S	S	S	S	R	R	S	S	S	S	R	S	S	S	S	S	S	S	S	S	S	S	S	ND	S	S	ND
WH006AW	++	R	R	S	S	S	S	R	R	S	S	R	R	R	R	R	R	R	S	S	S	S	S	S	S	S	R	—	ND	S	S	ND
WH017KL	+	S	S	S	S	S	S	S	S	S	S	S	S	S	S	R	S	S	S	S	S	S	S	S	S	S	S	S	ND	R	R	ND
WH026NS	Neg	ND	S	ND	S	ND	S	ND	S	ND	S	ND	S	ND	S	S	ND	S	ND	S	S	ND	S	S	ND	S	ND	S	ND	ND	R	ND
WH036ES	Neg	S	S	S	S	S	S	S	S	S	S	R	R	S	S	R	S	S	S	S	S	S	S	S	S	S	S	S	ND	S	S	ND
WH037PD	++	—	S	—	R	—	S	R	R	R	R	S	S	S	S	R	S	S	S	S	S	S	S	S	S	S	S	S	ND	S	S	ND

a S, susceptible.

b ND, sample or result not available.

c —, low sequence read coverage at position.

d R, resistant.

### Mixed infections and minority variants.

No mixed infections were detected. Using data normalized for read depth, from 32 matched sputa-MGIT samples, minor frequency variation was low, with only 88 minority biallelic sites meeting the quality criteria identified in all samples, representing 0.002% unique variable positions across the genomes. We undertook stringent procedures to exclude sequencing error, the presence of closely related M. tuberculosis complex species in sputum, contamination in and between sequencing runs, and lab contamination after sample collection as potential causes for the findings. None of the variant alleles were at positions known to be associated with antimicrobial resistance. In 41% (13) of cases, directly sequenced sputa had higher numbers of novel minority variants identified than the matched MGIT samples, compared to 19% (6) of MGIT samples with more minority variants in the sputum samples (see Fig. S4).

To control for the potential influence of the SureSelectXT step, we analyzed the proportions of minority variants shared between sputum and eight SureSelectXT enriched MGIT samples (40%), comparing between sputum and 16 nonenriched samples (38%), and found no statistically significant difference (*P* = 0.854). Overall, 37.2% of minority variants were concordant between sputum and MGIT samples, and the read frequencies with which they occurred were weakly correlated (see Fig. S5). This correlation was skewed by one patient (WH044IL), in whom both MGIT and sputum samples were more variable than other samples (Fig. S4) and in whom seven variants were at much higher frequencies in the MGIT than in sputum (at 30% to 45% versus ∼5%, respectively) (Fig. S5 and S6). However, there was no evidence of mixed genotypes. The five synonymous minor variants and two nonsynonymous variants occurring in two genes of unknown function (Rv3529c and Rv3888c) were distributed across the genome and were not shared across any other pairs of samples.

## DISCUSSION

We have shown that using target enrichment WGS methodology directly from diagnostic sputum samples generates resistance data, at most, up to 24 days earlier than MGIT culture WGS and up to 31 days faster than phenotypic testing of Mycobacterium tuberculosis. Sputum sequencing only achieved whole-genome sequences suitable for predicting resistance mutations in 32/43 (74%) samples, though this included a smear-negative sputum sample. Our demonstration that the quality of sequence data is strongly correlated with the input level of TB DNA (see Fig. S1 in the supplemental material) means that the success of sequencing can be predicted using semiquantitative methods such as smear microscopy and Xpert MTB/RIF (Fig. 2), notwithstanding their variable performance (25).

Our data compare well with a recent report describing WGS of sputum where contaminating human DNA had been depleted (26). While this method achieved a slightly faster turnaround time on diagnostic samples (2 versus 5 days), it may be less susceptible than targeted enrichment, as only 60% (24/40) of smear-positive samples yielded sequence data suitable for resistance prediction. The study did not report bacterial load, and so a thorough comparison of sensitivity cannot be performed. The methods are, however, highly complementary, and combining the two would likely improve the genome copy input and increase direct WGS sensitivity. Our experience with enrichment methods (27, 28) also predicts that a redesign of the first-generation probe set would further improve the detection of resistance mutations.

Direct sequencing of sputum is currently slower than rapid methods such as the Xpert MTB/RIF and the Hain MTBDRplus and MTBDRsl assays for detecting resistance. However, there are major advantages to using WGS. First, unlike existing rapid methods, it can accurately identify the precise nucleotide change causing resistance. In our study, Xpert MTB/RIF reported resistance in a susceptible organism where there was a nucleotide change at position 428 in the *rpoB* gene not associated with resistance (19–21). Where discordant resistance results were found between molecular methods and the routine phenotypic testing, we could not repeat the phenotypic testing, as this was carried out historically by a centralized reference laboratory. Therefore, these discrepancies cannot be confirmed, and this represents a limitation of this type of study. Second, WGS, unlike rapid methods which target specific mutations, is able to detect resistance mutations for a wider range of second- and third-line drugs and also new drugs where current rapid tests would require costly redesign. This has already helped us to personalize treatment in a case of drug-resistant M. tuberculosis (29). Third, the data from direct WGS of sputum can report evolutionary relationships between samples (Fig. S2), providing the most detailed information on transmission dynamics available.

An important objective of our study was to evaluate the potential for direct sequencing from sputum to detect mixed M. tuberculosis infections, which are suboptimally identified by MGIT and solid culture (13, 14). Mixed infections are important in the pathology of TB, and the ability to detect resistance variants that are not at a consensus level, although not necessarily common (30), can affect antibiotic stewardship (31). Mixed infections and MDR-TB are more prevalent in countries with a much higher burden of TB than the United Kingdom and a greater prevalence of drug resistance, while high levels of HIV amplify the problem (32, 33). We were able to detect significantly more minority single nucleotide variants (SNVs) in sputum compared to the matched MGIT sequence (Fig. S5), despite the mostly clonal populations in this study and the greater read depths achieved from MGIT sequencing. The origin of this heterogeneity remains unconfirmed, although we rigorously excluded contamination and methodological error. SNVs could be due to the presence in sputum of nontuberculous mycobacteria and other species which are known to have sequence homology with M. tuberculosis and may theoretically be detected by targeted enrichment. However, our use of highly stringent sequence mapping and the fact that the SNVs were detected across the genome and not concentrated in regions generally associated with cross hybridization suggest that they are real. In case WH044IL, seven SNVs present in sputum increased in frequency in the MGIT culture, possibly reflecting a selective growth advantage for this haplotype, particularly as one nonsynonymous SNV occurred in the Rv3888c gene, which has been shown to be essential for mycobacterial *in vitro* growth (34). This result confirms suggestions that diversity is lost and that culture-related selection of some variants can occur even during limited MGIT culture. Thus, MGIT culture may not be representative of the original sample and could potentially reduce the likelihood of identifying low-level resistance mutations and mixed infections that may act as a reservoir for resistance development.

The standard diagnostic workflows for M. tuberculosis are costly and time consuming. Ground breaking work within Public Health England has demonstrated that sequencing M. tuberculosis whole genomes from positive MGIT cultures is faster and less expensive (6), and where the sputum pathogen DNA concentration is low, early MGIT sequencing could still be the best possible workaround (15). Direct sequencing of M. tuberculosis from sputum has the potential to reduce the time to antimicrobial resistance detection within a clinically relevant time frame (26). We show here that its success is critically dependent on the input genome copies of pathogen DNA. While enrichment increases the cost of pathogen sequencing, this could be offset, as demonstrated in our study, by only enriching areas of interest on the genome. It is important to note that the infrastructure and expertise for rapid, high-throughput, targeted enrichment sequencing directly from clinical material of M. tuberculosis and other pathogens already exist in genomic centers where cancer and genetic disease sequencing uses this methodology. We believe, therefore, that effective scale-up and implementation of this rapid and accurate technology is relatively easy, once the technique has been optimized.

## Supplementary Material

Supplemental material
