# Supplementary material for: Direct Whole-Genome Sequencing of Sputum Accurately Identifies Drug-Resistant Mycobacterium tuberculosis Faster than MGIT Culture Sequencing
Source: J Clin Microbiol. 2018 Jul 26;56(8):e00666-18. doi: 10.1128/JCM.00666-18 (PMC6062781; doi:10.1128/JCM.00666-18)
Supplement: Supplemental material [file supp_56_8_e00666-18__index.html]

Supplemental material 

# Direct Whole-Genome Sequencing of Sputum Accurately Identifies Drug-Resistant Mycobacterium tuberculosis Faster than MGIT Culture Sequencing

## Supplemental material

- Supplemental file 1 -

  Table S1 (The 10 single nucleotide variants that were discordant at the consensus level between sputum and MGIT samples of nine patients) and Fig. S1 (Effect of genome copy input on median depth of coverage recovered for each sample and box plot showing difference in estimated genome copies available for library preparation for sputa compared to MGIT cultures), S2 (Maximum likelihood tree of whole genomes from paired sputum and MGIT samples from the same patients), S3 (Resistance mutations identified between samples sequenced with the whole-genome RNA bait set and the reduced bait set targeting only genes with resistance mutations), S4 (Histogram of minor-variant count in each sample for both MGIT and sputum samples and box plot showing number of unique minor variants present in either the MGIT or the sputum sample), S5 (Correlation of minor-variant read frequencies for shared variants between pairs of samples from the same individual), and S6 (Correlation of minor-variant read frequencies for shared variants between pairs of samples stratified by individual patients)

  PDF, 906K
