# Supplementary material for: Direct Whole-Genome Sequencing of Sputum Accurately Identifies Drug-Resistant Mycobacterium tuberculosis Faster than MGIT Culture Sequencing
Source: J Clin Microbiol. 2018 Jul 26;56(8):e00666-18. doi: 10.1128/JCM.00666-18 (PMC6062781; doi:10.1128/JCM.00666-18)
Supplement: Supplemental material [file JCM.00666-18_zjm999096043s1.pdf]

Supplementary materials

Supplementary table 1. The ten single nucleotide variants that were discordant at consensus level between sputum and MGIT samples of nine patients.

				**Sputum**			**MGIT**		
**Patient**	Position in H37Rv	Reference base	Variant base	Total read depth	Variant read depth	Variant read frequency	Total read depth	Variant read depth	Variant read frequency
BH052SA	2339240	G	A T	345	150	43.5	59	37	62.7
BH056ESDS	2945167	G		345	211	61.2	65	27	41.5
RF001SH	2339240	G	A	267	202	75.7	197	62	31.5
RF002AH	1415643	G	A	105	55	52.4	604	283	46.9
RF003JV	2262026	T	G	44	25	56.8	128	52	40.6
RF017MW	4317344	C	T	145	61	42.1	239	138	57.7
RF021RC	2639192	G	A	290	157	54.1	112	41	36.6
WH017KL	1096633	T	G	53	25	47.2	392	216	55.1
	2945167	G	T	72	33	45.8	487	266	54.6
WH035CL	1481337	G	A	102	59	57.8	169	77	45.6

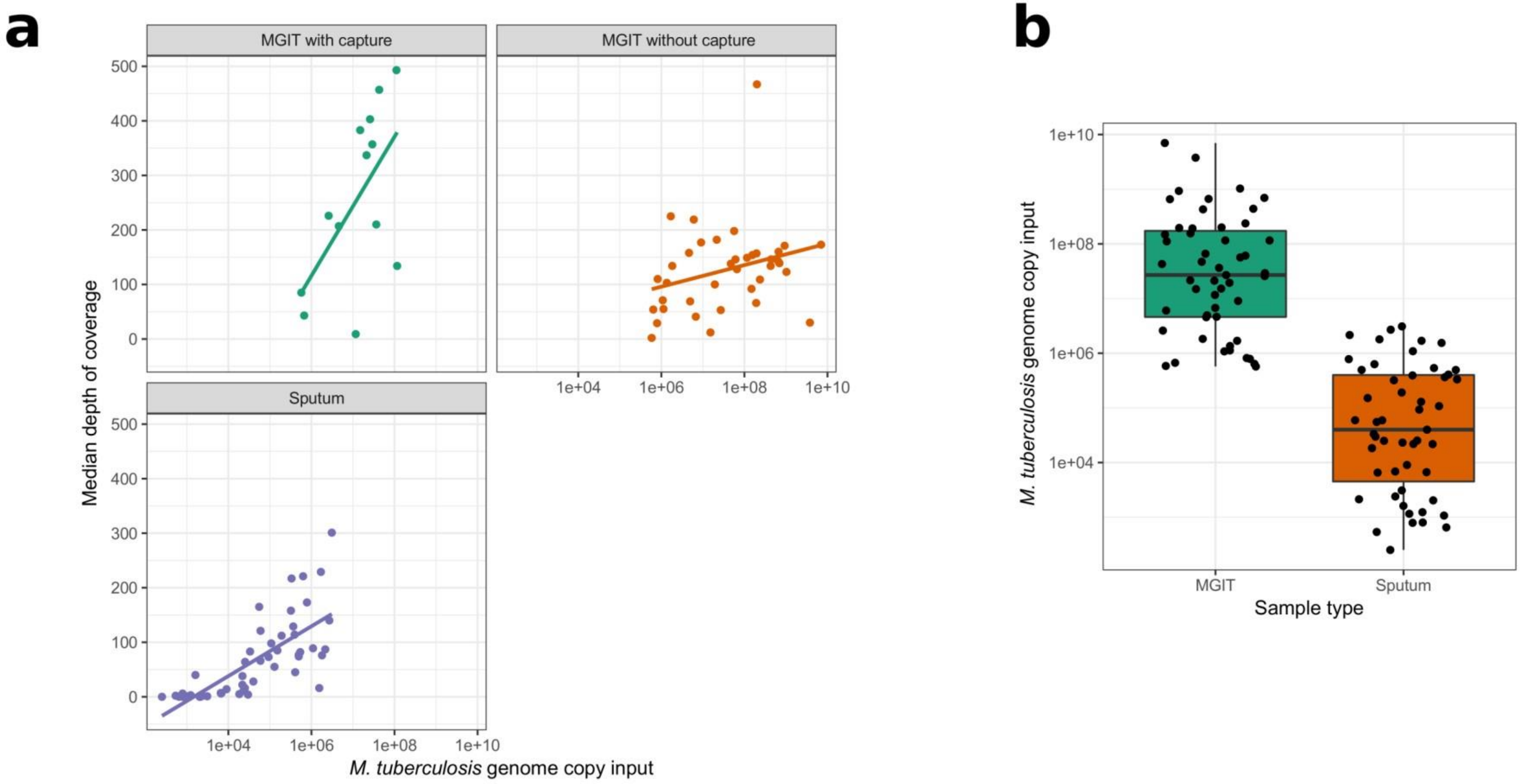

Supplementary figure 1. (A) Effect of genome copy input on the median depth of coverage recovered for each sample stratified by whether sample was prepared from sputum using SureSelectXT (Sputum), MGIT with SureSelectXT (MGIT with capture) or MGIT with NebNext Ultra II (MGIT without capture). Each line plotted through the points represents linear regression of coverage against genome copy input. (B) Boxplot showing difference in the estimated genome copies available for library preparation for sputa compared to MGIT cultures.

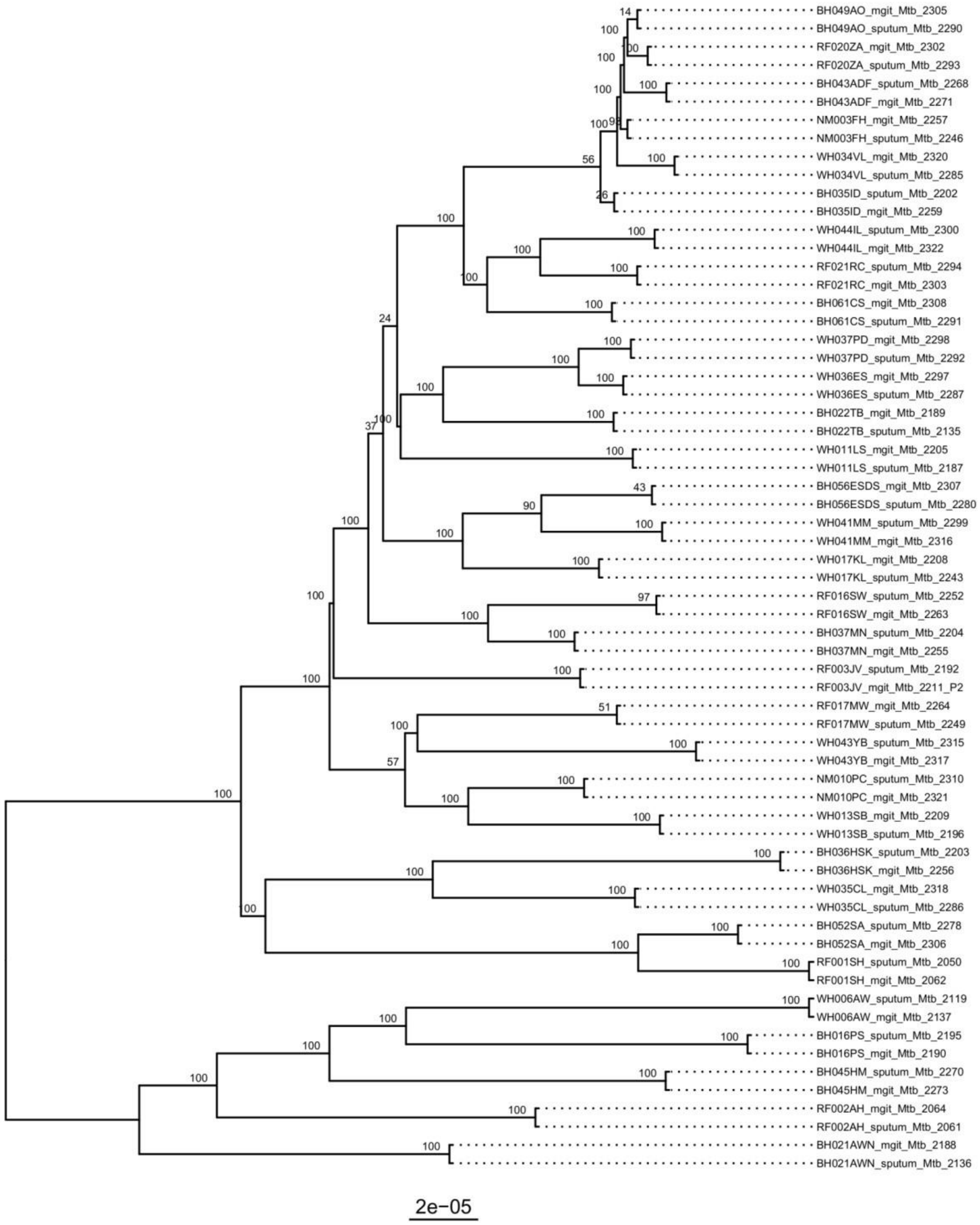

Supplementary figure 2. Maximum likelihood tree of whole genomes from paired sputum and MGIT samples from the same patients. Bootstrap confidence values are reported from 99 replicates and the tree is midpoint rooted. Scale bar represents the number of substitutions per site.

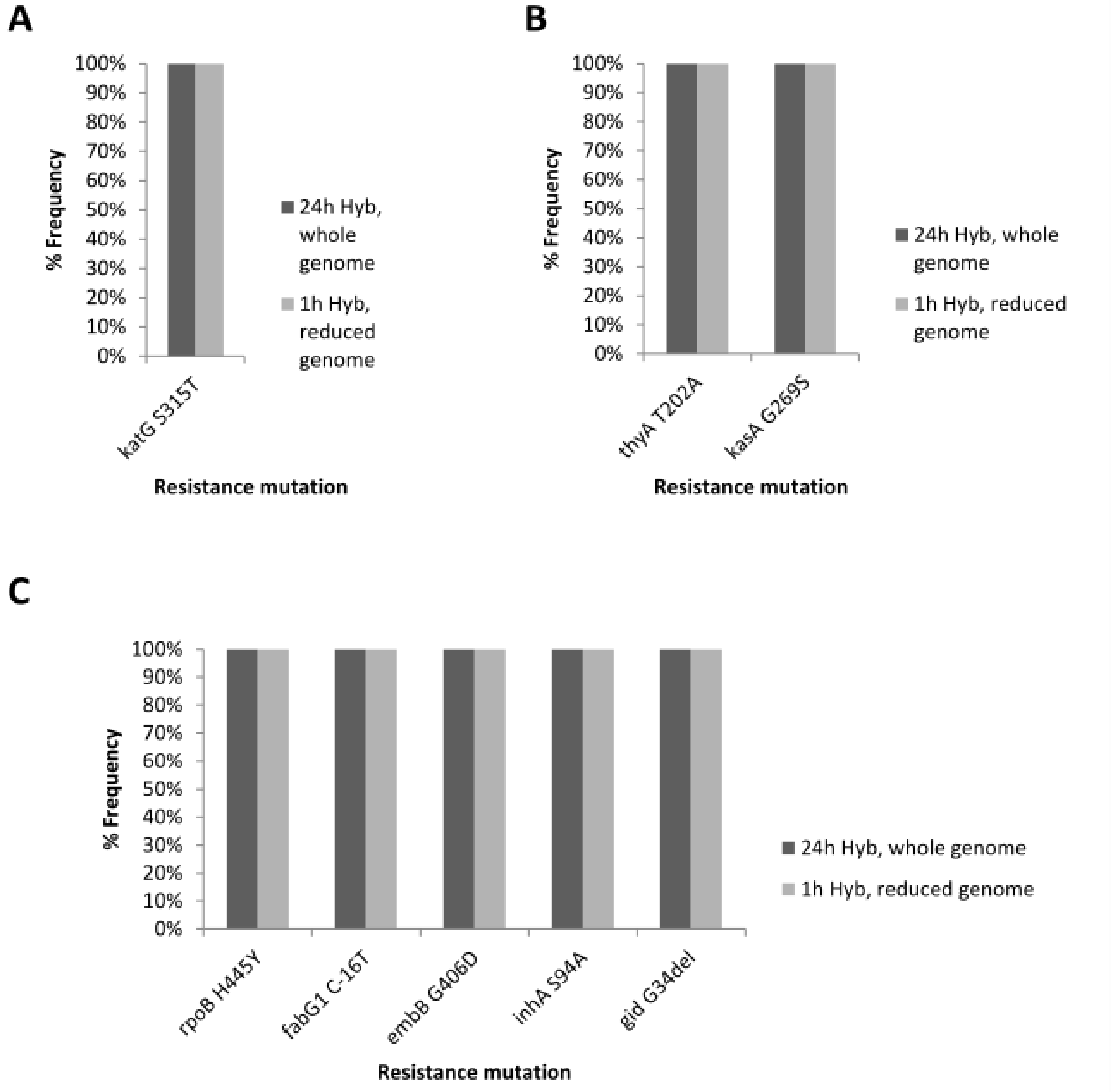

Supplementary figure 3. Resistance mutations identified between the samples sequenced with the whole genome RNA bait set (24h Hyb, whole genome) and the reduced bait set that only targets genes with resistance mutations (1h Hyb, reduced genome). The read frequency at each resistance site is plotted for patients (A) RF002AH, (B) BH001MC and (C) WH006AW.

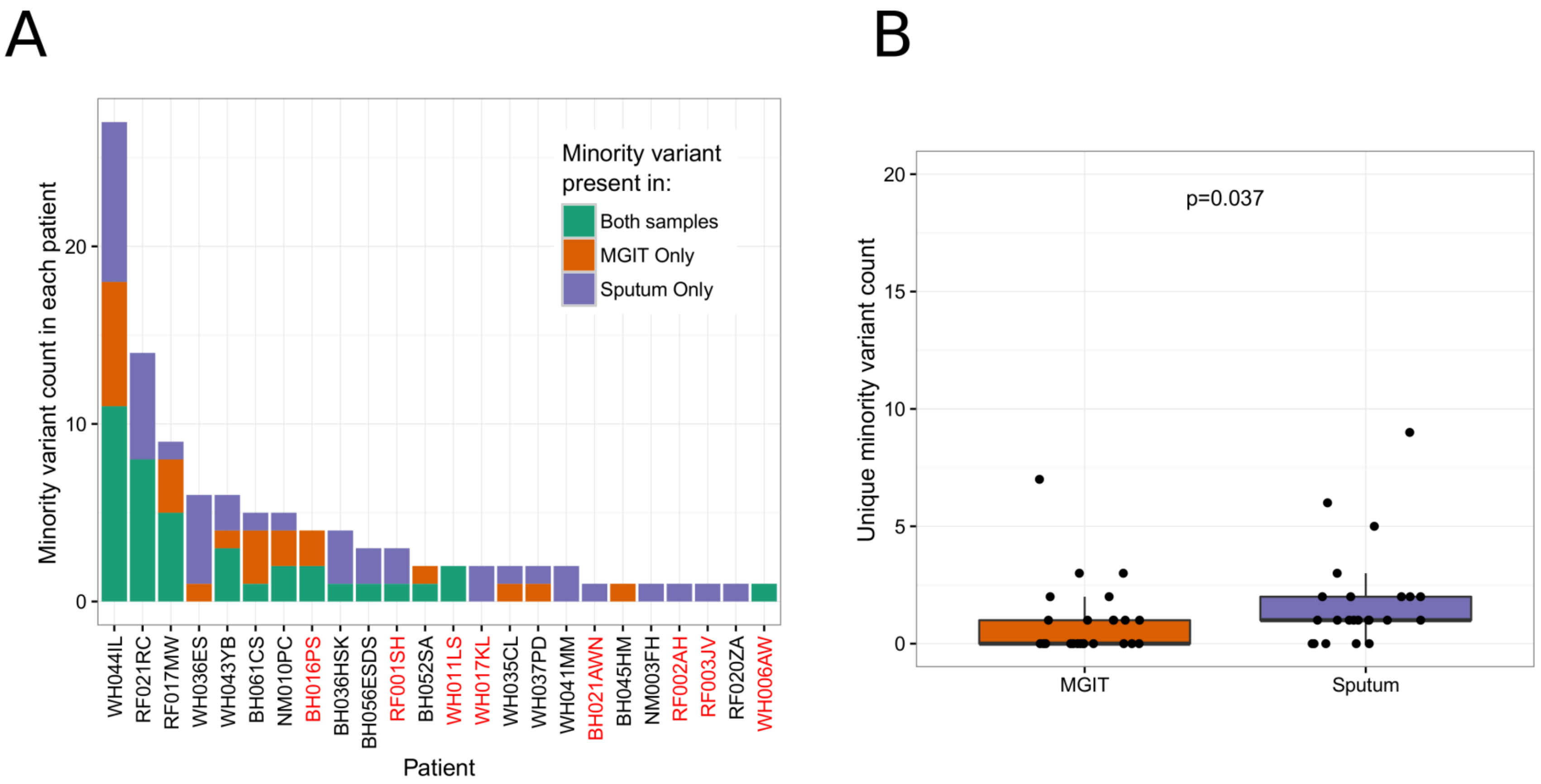

Supplementary figure 4. (A) Histogram of the minor variants count in each sample for both MGIT and sputum samples. Bars are coloured as to whether the variant is shared in both paired samples or is unique to the MGIT or sputum sample. Patient identifiers are highlighted red if MGIT sample was prepared using SureSelectXT enrichment and in black if they were prepared without enrichment. (B) Boxplot showing the number of unique minor variants present in either the MGIT or sputum sample.

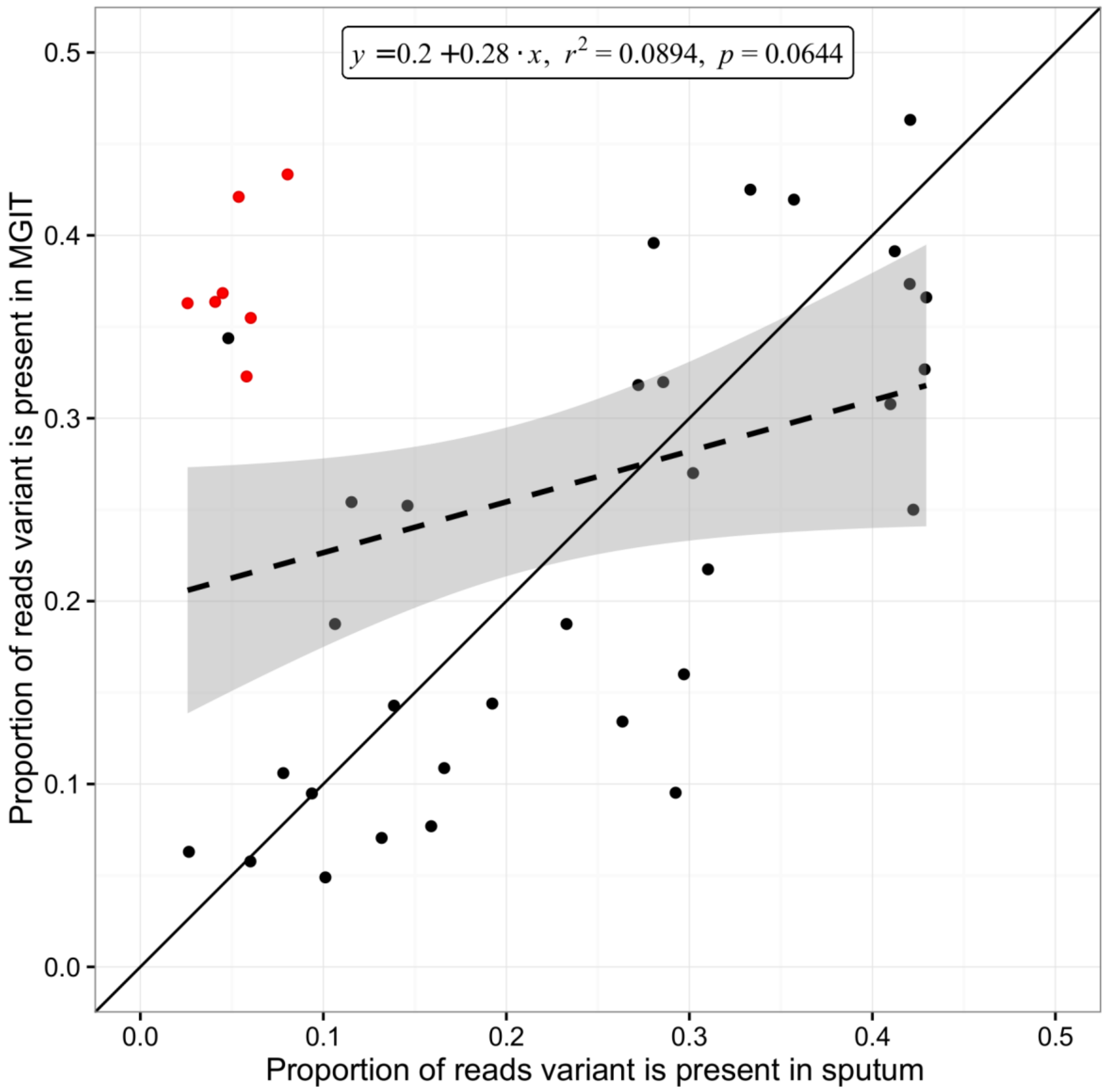

Supplementary figure 5. Correlation of minor variant read frequencies for shared variants between pairs of samples from the same individual. All individuals are plotted in the same graph with shared variants from patient WH044IL highlighted in red.

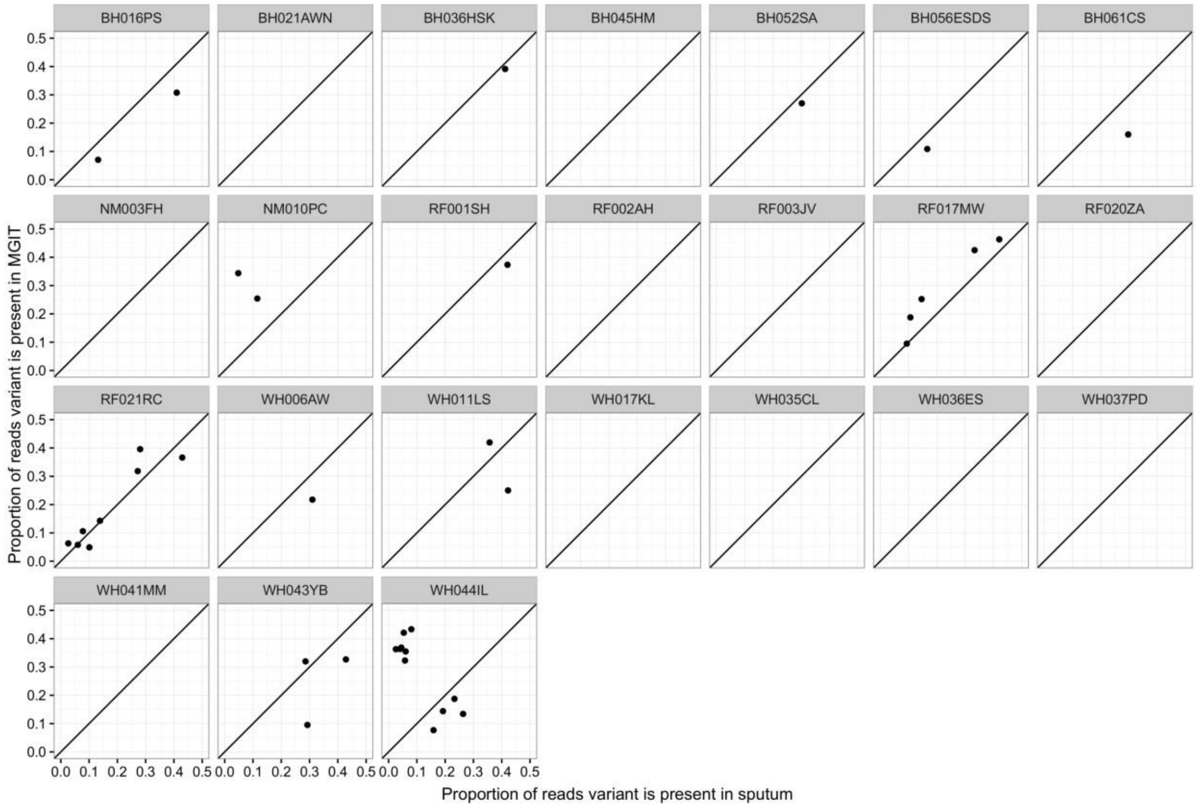

Supplementary figure 6. Correlation of minor variant read frequencies for shared variants between pairs of the samples as from figure 6 but instead stratified by individual patients.
